# Optimal Acquisition Number for Hepatic Shear Wave Velocity Measurements in Children

**DOI:** 10.1371/journal.pone.0168758

**Published:** 2016-12-21

**Authors:** Hyun Joo Shin, Myung-Joon Kim, Ha Yan Kim, Yun Ho Roh, Mi-Jung Lee

**Affiliations:** 1 Department of Radiology and Research Institute of Radiological Science, Severance Children's Hospital, Seoul, Korea; 2 Biostatistics Collaboration Unit, Yonsei University College of Medicine, Seoul, Korea; Medizinische Fakultat der RWTH Aachen, GERMANY

## Abstract

**Objective:**

To investigate the minimum optimal acquisition number of hepatic shear wave velocities (SWVs) on ultrasound elastography in children.

**Materials and Methods:**

We prospectively performed hepatic supersonic shear wave elastography in children of four groups (group A-C, healthy children, group A with 0–5 years old; group B with 6–10 years old; group C with 11–18 years old; and group D, children with previous Kasai operation) with free breathing (FB) and breath holding (BH) status, if possible. SWVs were measured fifteen times for each child at a 4 cm depth for the right lobe using a 1–6 MHz convex transducer. Mean SWVs from three, five, and seven acquisitions were compared to the mean SWV from fifteen measurements, using an intraclass correlation coefficient (ICC) analyzed with the 1,000 times bootstrap method.

**Results:**

Total eighty-eight children were included (25 children in group A, 30 children in group B, 21 children in group C, and 12 children in group D). The mean SWVs from fifteen measurements in FB status were 5.5 ± 1.3 kPa for groups A-C together and 8.0 ± 2.2 kPa for group D. For all groups together, mean SWVs from the three (ICC 0.944 and 0.937), five (ICC 0.958 and 0.938) and seven (ICC 0.969 and 0.941) acquisitions demonstrated almost perfect agreement with the reference of fifteen acquisitions in both FB and BH status, respectively. A subgroup analysis showed three measurements were in almost perfect agreement during FB for groups B-D and strong agreement (ICC 0.675) for group A.

**Conclusion:**

Three acquisitions can be enough for hepatic SWVs in children more than 6 years old regardless of breathing status or hepatic pathology. More acquisitions are recommended for children under the age of 5 years during FB.

## Introduction

In children, hepatic fibrosis can result from various conditions including hepatitis from viral infections or medications, biliary disease from biliary atresia, nonalcoholic steatohepatitis, metabolic disease such as Wilson’s disease, and vascular alteration such as congestive heart failure [1]. It is important to accurately diagnose the degree of hepatic fibrosis to reduce patient morbidity and mortality from the progression of liver damage. Although liver biopsy is the standard reference for diagnosis of hepatic fibrosis, its invasive nature and sampling errors are serious shortcomings because a biopsy specimen cannot represent the whole liver [2,3]. Therefore, many efforts have been made to evaluate the degree of hepatic fibrosis by noninvasive imaging techniques [3].

One of these imaging techniques is ultrasound elastography. This method is used to measure tissue stiffness after stress or shear waves are applied to the region of interest [4]. Ultrasound elastography using shear waves can help physicians measure tissue stiffness quantitatively by simply putting regions of interest (ROIs) on gray scale images during real time ultrasound examinations.

The Society of Radiologists in Ultrasound recently published a consensus statement on the technical aspects of ultrasound elastography for the evaluation of hepatic fibrosis [3]. They recommended ten measurements at the same hepatic location. However, they questioned the necessity of taking ten repeated measurements, especially for good-quality examinations [3]. Moreover, the number of hepatic shear wave velocity (SWV) measurements acquired varied from three to twenty in previous studies [5–11].

There are no specific manufacturer recommendations on how many measurements are adequate to obtain reliable results. In addition, repeating procedures to obtain measurements ten times is difficult to do in young children. Even though a child may be calm at the beginning of an examination, obtaining reliable and stable results becomes more difficult as the examination time increases. In addition, young children cannot hold their breath repetitively and regularly during SWV acquisition. Therefore, we investigated the optimal and minimum acquisition number of hepatic SWVs for ultrasound elastography in children according to their breathing methods.

## Materials and Methods

### Subjects

Our Institutional Review Board approved this prospective study. For the evaluation of the optimal acquisition number in normal liver of children, we invited healthy children under the age of 18 years old who wanted to undergo hepatic ultrasound elastography for check-up from March 2015 to August 2016 at our institution. All subjects and their parents agreed to the process and signed informed consent. All patients fasted for four hours prior to the examination. We excluded children who had a medical history of liver disease, abnormal ultrasound results prior to the elastography examination, or who did not cooperate during ultrasound elastography. For the evaluation of the optimal acquisition number in children with liver disease, we included children who had undergone a Kasai operation due to biliary atresia during the same study period. We excluded children who did not cooperate during the ultrasound elastography from them. We divided the children into four groups; group A (healthy children 0–5 years old), group B (healthy children 6–10 years old), group C (healthy children 11–18 years old), and group D (children who had undergone a Kasai operation).

### Ultrasound elastography

**Fig 1:**
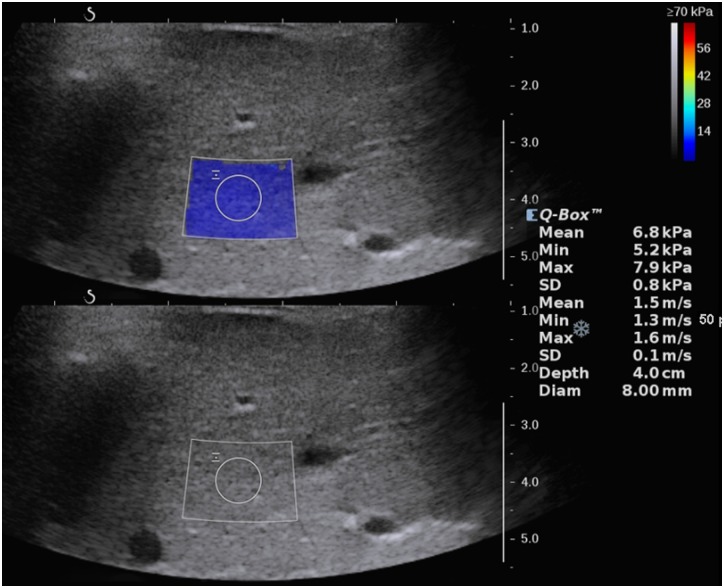
Ultrasound elastography image obtained from the liver of a 6-year-old girl. The circular region of interest was placed on the color-coded map of homogeneous liver parenchyma at a location that avoided vessels and bile ducts in the right lobe. The child was in the supine position with free breathing status. Mean values were simultaneously provided for both units of elasticity and velocity as 6.8 kPa and 1.5 m/sec.

Ultrasound elastography examinations were performed using supersonic shear wave elastography (SWE, Aixplorer, SuperSonic Imagine, Aix-en-Provence, France; software version of 9.2) with a 1–6 MHz convex transducer (model number of SC6-1). Examinations were performed by one of two experienced pediatric radiologists. Prior to SWE examination, routine abdominal ultrasound was performed to exclude children who might have any abnormalities in the abdomen, including the liver. After that, hepatic SWVs were obtained while the children were in the supine position with regular free breathing (FB) status. The transducer was held at a perpendicular angle to the skin. We used a right intercostal approach and obtained hepatic SWVs fifteen times from fifteen color-coded maps at the right lobe, avoiding hepatic vessels and bile ducts, at a 4 cm depth from the dermis. A round ROI with an 8 mm diameter (the automatically set size for routine abdominal study for SWE) was placed on each color-coded map of elasticity in the targeted liver (Fig 1). The machine automatically provided values in units of both kPa and m/sec simultaneously. We recorded the mean value of the ROI in the unit of kPa for each acquisition. When the children were able to hold their breath for a moment, hepatic SWVs were obtained fifteen times repeatedly in breath hold (BH) status using the same method above.

### Statistical analysis

Statistical analyses were performed using SPSS version 20.0.0 (IBM Corp., Armonk, NY, USA). The mean SWV from fifteen measurements was considered as the reference standard for hepatic elasticity in each child. To evaluate the strength of agreement between a smaller number of measurements and the fifteen measurements, we used a 1,000 times bootstrap method with repeated random sampling for three, five, and seven acquisitions. We calculated mean values and standard deviations for each acquisition. We also calculated the intraclass correlation coefficient (ICC) and 95% confidence interval (CI) for each number of acquisition in FB and BH status of four groups.

Strength of agreement was classified by ICC value as follows: 0.00–0.20, poor; 0.21–0.40, fair; 0.41–0.60, moderate; 0.61–0.80, strong; and 0.81–1.00, almost perfect.

## Results

During the study period, total 88 children (M:F = 45:43) were included in this study. Among them, 25 children in group A (M:F = 18:7, mean age 2.6 ± 1.8 years), 30 children in group B (M:F = 10:20, mean age 7.3 ± 1.2 years), 21 children in group C (M:F = 14:7, mean age 13.4 ± 2.4 years), and 12 children in group D (M:F = 3:9, mean age 9.3 ± 4.4 years, age range 3–18 years old) were included in this study. No patient was excluded due to incidental abnormal findings during ultrasound examination or for noncooperation in groups A-C. In group D, all of the children underwent ultrasonography examination for the routine follow-up without clinical evidence of acute illness including cholangitis, and there was no incidental mass or cystic lesion in the liver.

Mean SWV and standard deviations for the three, five and seven measurements were calculated using a 1,000 times bootstrap method for each subject in units of kPa as shown in S1 Table. Table 1 shows the results for ICC comparisons in each group. The mean SWV from fifteen measurements in FB status of the groups A-C together was 5.5 ± 1.3 kPa. The mean SWV from fifteen measurements in FB status of the group D was 8.0 ± 2.2 kPa. The results showed no specific tendency of increased or decreased standard deviation according to the measurement number or to the breathing method in this study. There was no technical measurement failure when obtaining hepatic SWVs, even in FB status.

**Table 1:** Intraclass correlation coefficient (ICC) comparison in four groups.

Number of measurements	Breathing method	Group A (0–5 years)	Group B (6–10 years)	Group C (11–18 years)	Group D (diffuse liver disease)
3	FB	0.675 (0.376, 0.834)	0.932 (0.885, 0.966)	0.936 (0.829, 0.985)	0.971 (0.880, 0.997)
5		0.782 (0.601, 0.892)	0.942 (0.896, 0.975)	0.954 (0.881, 0.988)	0.971 (0.892, 0.995)
7		0.854 (0.707, 0.940)	0.953 (0.907, 0.982)	0.959 (0.892, 0.989)	0.977 (0.933, 0.990)
3	BH		0.937 (0.819, 0.988)	0.904 (0.789, 0.969)	0.863 (0.585, 0.996)
5			0.938 (0.833, 0.986)	0.943 (0.885, 0.980)	0.815 (0.485, 0.995)
7			0.927 (0.821, 0.983)	0.954 (0.907, 0.984)	0.808 (0.421, 0.993)

FB, free breathing; BH, breath holding

Values are presented as ICC (95% confidence interval).

In all children from four groups, three measurements (ICC, 0.944; 95% CI, 0.899–0.972), five measurements (ICC, 0.958; 95% CI, 0.923–0.978) and seven measurements (ICC, 0.969; 95% CI, 0.945–0.982) demonstrated almost perfect agreement with the reference of fifteen measurements in FB status. In BH status, three measurements (ICC, 0.937; 95% CI, 0.887–0.978), five measurements (ICC, 0.938; 95% CI, 0.876–0.981), and seven measurements (ICC, 0.941; 95% CI, 0.878–0.983) also demonstrated almost perfect agreement with the reference of fifteen measurements. In subgroup analysis (Table 1), the ICC values for three measurements (ICC, 0.675; 95% CI, 0.376–0.834) and five measurements (ICC, 0.782; 95% CI, 0.601–0.892) showed strong agreement in FB status of group A. The ICC value of seven measurements in group A was 0.854 (95% CI, 0.707–0.940), indicating almost perfect agreement. The ICC values in BH status of group A could not be calculated because a few children could sustain their breath in this age group (only four children were possible). In addition, the other measurements including three, five, and seven measurements from groups B, C, and D showed almost perfect agreement with the fifteen times of acquisition, with ICC values of more than 0.8 in both FB and BH status. The results showed that the breathing status and the liver disease status did not affect overall measurement agreements even for the three times acquisition.

## Discussion

Recent studies have summarized well-known optimal conditions for the measurement of elasticity values using ARFI and SWE [3,12]. These included fasting for 4–6 hours, staying in the supine or slight left lateral decubitus position with elevation of the right arm to allow easier access to the right intercostal space, shallow BH for a few seconds, and placing the ROI in the right hepatic lobe perpendicular to the liver capsule below at a 2.0 cm depth (typically located 4-5cm below the transducer), and avoiding large hepatic vessels, bile ducts and rib shadows [3,12]. Ten repeated measurements have generally been accepted as the appropriate number for adults in those studies [3,12]. However, there are various reports concerning the reproducibility of acquisition numbers for ultrasound elastography. One study recommended at least 50 supervised measurements for constant results [13]. Contrary to that result, another study demonstrated that only one measurement might be sufficient for accurate diagnosis of hepatic fibrosis [14]. The other studies commented that repeating measurements three or five times allowed reliable assessment of hepatic stiffness [5,15].

The World Federation for Ultrasound in Medicine and Biology recommended 5 to 10 measurements for acoustic radiation force impulse (ARFI) imaging and 4 measurements for supersonic SWE [12,16]. The Society of Radiologists in Ultrasound published a consensus statement that 10 measurements obtained in the same location was the best practice for performing ultrasound elastography [3]. However, it is not easy to measure tissue elasticity ten times in pediatric patients, especially in young children. Repetitive BH is challenging for these patients, even if it is only for a few seconds. Moreover, with longer exams, patient cooperation can become a significant issue in children. Therefore, to best utilize ultrasound elastography in pediatric patients, it is essential to know the minimum number of acquisitions needed to obtain the optimal elasticity value.

We tried to evaluate the optimal acquisition number for regular FB status in children. We found that three acquisitions in FB status showed almost perfect agreement with fifteen acquisitions regardless of the presence of liver disease in children more than 6 years old. In addition, variation was not remarkably different between FB and BH status, except for children younger than 5 years old who could not hold their breath and showed strong agreement in three and five measurements in FB status though. Therefore, we suggest that a three-time acquisition of tissue elasticity in FB status is enough to measure SWVs in children more than 6 years old, where good-quality measures of stiffness are obtained with regular FB status, i.e., without BH. In addition, more than three measurements and about seven measurements could be reasonable for children under the age of 5 years, because the ICC value in this study showed almost perfect agreement with seven measurements for this age group. Ferraioli et al. [16] recommended four measurements for supersonic SWE and explained that these measurements were enough because one displayed image during supersonic SWE represented roughly three averaged frames in time due to temporal persistence. However, to our knowledge, this is the first study to objectively demonstrate an optimal acquisition number for SWE in children with FB status.

For pediatric patients, several conditions have to be considered when utilizing ultrasound elastography. First, the acquisition number chosen is important because it directly affects the duration of the examination. The duration for SWV acquisition has been measured as 5 minutes for 10 repeated measurements and 8–14 minutes for 20 repeated measurements in adult studies [9,13]. When SWVs are measured in children with the same number of repeated measurements as in adults, more time is required. Second, the necessity of the BH technique should be questioned because it is not easy for children to hold their breath repetitively, and it can be impossible for young children. In adult studies, patients who could not sustain their breath adequately were excluded because of motion artifact concerns [17,18]. However, doing so is not possible in pediatric examinations as the number of patients who would be excluded would be significant. Thus, several pediatric studies have performed examinations with FB acquisition during ultrasound elastography [6,19,20]. One pediatric study mentioned that breathing and weak movement did not interrupt valid data capture during ultrasound elastography [19]. Another study used a three-time SWV measurement for abdominal solid organs in children with FB status [6]. Researchers in this study worried that this method could result in increased variability of SWV compared to adult studies performed with BH status [6]. It should also be noted that the rhythm of breathing can become more irregular and variable after a trial of BH in children. Therefore, long examination times and the need for BH can be important limitations for ultrasound elastography in children.

Our study is important because we objectively demonstrated that variability from FB was not significant. In addition, our data showed that even three repeated acquisitions in FB status demonstrated almost perfect agreement with fifteen repeated acquisitions for SWV measurements in children more than 6 years old regardless of hepatic pathology. We also found strong measurement agreement in children under the age of 5 years.

There are several limitations in this study. First, we could not analyze ICC values in BH status for group A, because only a few children could hold their breath. However, our result is valuable because we demonstrated excellent agreement between three and fifteen measurements in FB status in these young children, because BH is frequently impossible in this age group in real practice. Second, the number of children with liver disease was small, and the degree of liver stiffness was not diverse, showing a standard deviation of 2.2 kPa in group D. A recent adults’ study found that the values from different numbers of acquisition were different for fatty liver disease and for liver stiffness higher than than 10 kPa [15]. Results could also be different for children, if liver stiffness is heterogeneous or higher than that in our study. At last, we used only one ultrasound elastography machine. This machine is known to have a very fast acquisition speed of ultrasound images, of at least 5,000 to 20,000 frames per second [21]. Such a fast acquisition could have reduced the risk of artifacts introduced by patient or investigator movement. If a different machine such as pointed SWE or a different transducer was used, or if different acquisition depths and ROI sizes were applied, the agreement of SWVs might be affected, and the optimal acquisition numbers found under different conditions would vary from our results. Thus, further studies are needed to evaluate the minimum optimal acquisition number required under such varying conditions.

## Conclusions

Our study demonstrated almost perfect agreement in SWVs obtained from three, five, and seven measurements when compared with the values from fifteen measurements in children more than 6 years old regardless of breathing or liver disease status. Three and five measurements showed strong agreement and seven measurements showed almost perfect agreement with fifteen measurements during FB in the youngest children (group A). Therefore, we suggest that a three-time acquisition in FB status appears to be adequate for measurement of SWVs in children more than 6 years old, when good-quality measures of stiffness are obtained for regular FB status. In addition, more than three measurements and about seven measurements seem to be reasonable for children under 5 years during FB. The reduced number of acquisitions could shorten examination times and make examinations easier for children without the need for BH, thus result in wider application of ultrasound elastography in children.

## Supporting Information

S1 Table Mean shear wave velocities (SWVs) and standard deviations for three, five, seven and fifteen measurements using a 1,000 bootstrap methods. (DOCX)
