# Supplementary material for: Optimal Acquisition Number for Hepatic Shear Wave Velocity Measurements in Children
Source: PLoS One. 2016 Dec 21;11(12):e0168758. doi: 10.1371/journal.pone.0168758 (PMC5176183; doi:10.1371/journal.pone.0168758)
Supplement: S1 Table — (DOCX) [file pone.0168758.s001.docx]

**S1 Table. Mean shear wave velocities (SWVs) and standard deviations for three, five, seven and fifteen measurements using a 1,000 bootstrap methods.**

1. Group A (0-5 years)

| Subjects | Breathing  methods | 3 | 5 | 7 | 15 |
| --- | --- | --- | --- | --- | --- |
| 1 | FB | 4.97 ± 0.06 | 4.98 ± 0.04 | 5.26 ± 1.38 | 6.29 ± 1.6 |
| 2 | FB | 4.2 ± 0.1 | 4.24 ± 0.09 | 4.27 ± 0.1 | 4.34 ± 0.09 |
| 3 | FB | 4.57 ± 0.12 | 4.54 ± 0.09 | 4.53 ± 0.08 | 4.62 ± 0.15 |
| 4 | FB | 4.5 ± 0.1 | 4.54 ± 0.17 | 4.57 ± 0.15 | 4.62 ± 0.2 |
| 5 | FB | 4.5 ± 0 | 4.4 ± 0.41 | 4.23 ± 0.45 | 4.14 ± 0.39 |
| 6 | FB | 5.4 ± 0 | 5.32 ± 0.18 | 5.23 ± 0.21 | 4.82 ± 0.46 |
| 7 | FB | 7.2 ± 0 | 7.2 ± 0 | 6.8 ± 0.68 | 6.73 ± 0.52 |
| 8 | FB | 5.4 ± 0 | 5.36 ± 0.05 | 4.97 ± 0.67 | 5.91 ± 2.3 |
| 9 | FB | 4.8 ± 0 | 4.8 ± 0 | 4.8 ± 0 | 5.07 ± 1.04 |
| 10 | FB | 3.7 ± 0 | 3.7 ± 0 | 3.7 ± 0 | 3.97 ± 0.35 |
|  | BH | 5.2 ± 0 | 5.2 ± 0 | 5.2 ± 0 | 5.15 ± 0.18 |
| 11 | FB | 5.53 ± 0.58 | 4.6 ± 1.34 | 4.2 ± 1.29 | 3.83 ± 0.96 |
| 12 | FB | 4.9 ± 0 | 4.9 ± 0 | 4.9 ± 0 | 5.06 ± 0.56 |
|  | BH | 2.9 ± 0 | 2.9 ± 0 | 2.9 ± 0 | 2.87 ± 0.13 |
| 13 | FB | 7 ± 0 | 6.74 ± 0.58 | 6.44 ± 0.69 | 6.02 ± 0.65 |
| 14 | FB | 5.3 ± 0 | 5.3 ± 0 | 5.3 ± 0 | 4.9 ± 0.41 |
| 15 | FB | 5.1 ± 0 | 5.1 ± 0 | 5.07 ± 0.05 | 4.95 ± 0.16 |
| 16 | FB | 5.1 ± 0 | 4.98 ± 0.16 | 4.93 ± 0.16 | 5.23 ± 0.55 |
| 17 | FB | 5.83 ± 0.06 | 5.82 ± 0.04 | 5.81 ± 0.04 | 5.67 ± 0.26 |
| 18 | FB | 5.1 ± 0.17 | 5.14 ± 0.15 | 5.16 ± 0.17 | 5.3 ± 0.25 |
| 19 | FB | 4.23 ± 0.06 | 4.22 ± 0.04 | 4.03 ± 0.33 | 4.24 ± 0.86 |
| 20 | FB | 5.2 ± 0 | 5.2 ± 0 | 5.23 ± 0.05 | 5.37 ± 0.25 |
|  | BH | 6.2 ± 0 | 6.18 ± 0.04 | 6.07 ± 0.19 | 4.93 ± 1.15 |
| 21 | FB | 4 ± 0 | 4.24 ± 0.33 | 4.49 ± 0.58 | 5.32 ± 0.91 |
|  | BH | 6.4 ± 0 | 6.4 ± 0 | 6.4 ± 0 | 6.4 ± 0 |
| 22 | FB | 5 ± 0 | 5 ± 0 | 5 ± 0 | 5.2 ± 0.22 |
| 23 | FB | 5 ± 0.89 | 5.08 ± 0.7 | 4.9 ± 0.7 | 4.69 ± 0.64 |
| 24 | FB | 6.03 ± 0.55 | 6.14 ± 0.47 | 5.94 ± 0.6 | 5.65 ± 0.93 |
| 25 | FB | 8.83 ± 0.4 | 8.38 ± 0.8 | 7.7 ± 1.33 | 6.76 ± 1.33 |

1. Group B (6-10 years)

| Subjects | Breathing  methods | 3 | 5 | 7 | 15 |
| --- | --- | --- | --- | --- | --- |
| 1 | FB | 3.87 ± 0.06 | 3.88 ± 0.04 | 3.9 ± 0.06 | 3.81 ± 0.1 |
|  | BH | 3.93 ± 0.06 | 3.92 ± 0.04 | 3.96 ± 0.08 | 3.95 ± 0.09 |
| 2 | FB | 5.3 ± 0.1 | 5.26 ± 0.09 | 5.24 ± 0.08 | 5.22 ± 0.06 |
| 3 | FB | 5.53 ± 0.06 | 5.3 ± 0.32 | 5.34 ± 0.29 | 5.46 ± 0.25 |
|  | BH | 5.2 ± 0 | 5.2 ± 0 | 5.17 ± 0.05 | 5.15 ± 0.06 |
| 4 | FB | 4.63 ± 0.06 | 4.56 ± 0.15 | 4.76 ± 0.48 | 4.41 ± 0.8 |
|  | BH | 6 ± 0 | 6 ± 0 | 6 ± 0 | 5.48 ± 0.66 |
| 5 | FB | 3.5 ± 0 | 3.5 ± 0 | 3.46 ± 0.11 | 3.59 ± 0.69 |
|  | BH | 4.37 ± 0.23 | 4.5 ± 0.28 | 4.61 ± 0.3 | 4.36 ± 0.45 |
| 6 | FB | 4.1 ± 0 | 4.1 ± 0 | 4.1 ± 0 | 4.26 ± 0.15 |
|  | BH | 5.4 ± 0 | 5.4 ± 0 | 5.4 ± 0 | 5.27 ± 0.2 |
| 7 | FB | 6.6 ± 0 | 6.6 ± 0 | 6.6 ± 0 | 6.37 ± 0.7 |
|  | BH | 5.3 ± 0 | 5.3 ± 0 | 5.3 ± 0 | 5.42 ± 0.25 |
| 8 | FB | 6.2 ± 1.21 | 5.7 ± 1.1 | 6.1 ± 1.13 | 6.05 ± 1.08 |
|  | BH | 5.6 ± 0 | 5.6 ± 0 | 5.6 ± 0 | 5.73 ± 0.13 |
| 9 | FB | 4.5 ± 0 | 4.46 ± 0.05 | 4.39 ± 0.18 | 4.34 ± 0.14 |
|  | BH | 4.73 ± 0.15 | 4.7 ± 0.12 | 4.7 ± 0.12 | 4.55 ± 0.19 |
| 10 | FB | 4.5 ± 0 | 4.5 ± 0 | 4.5 ± 0 | 4.39 ± 0.16 |
|  | BH | 4 ± 0 | 4 ± 0 | 3.97 ± 0.05 | 4.28 ± 0.46 |
| 11 | FB | 4.8 ± 0 | 4.8 ± 0 | 4.79 ± 0.04 | 4.72 ± 0.1 |
|  | BH | 5.4 ± 0 | 5.4 ± 0 | 5.43 ± 0.05 | 5.36 ± 0.12 |
| 12 | FB | 5 ± 0.7 | 4.78 ± 0.63 | 4.91 ± 0.6 | 5.31 ± 0.72 |
| 13 | FB | 8.43 ± 1.62 | 8.06 ± 1.41 | 7.81 ± 1.22 | 6.82 ± 1.43 |
| 14 | FB | 5.6 ± 1.21 | 4.98 ± 1.22 | 5.4 ± 1.24 | 5.75 ± 1.35 |
| 15 | FB | 5.2 ± 1.21 | 4.16 ± 1.66 | 4.2 ± 1.39 | 5.47 ± 1.77 |
| 16 | FB | 6.37 ± 0.38 | 6.1 ± 0.47 | 6.19 ± 0.42 | 5.88 ± 0.54 |
| 17 | FB | 4.27 ± 0.93 | 4.62 ± 0.94 | 4.39 ± 0.89 | 4.41 ± 0.79 |
| 18 | FB | 7.93 ± 0.75 | 8.72 ± 1.23 | 8.9 ± 1.24 | 8.89 ± 1.76 |
| 19 | FB | 5.1 ± 0.4 | 5.46 ± 0.57 | 5.63 ± 0.57 | 5.45 ± 0.6 |
| 20 | FB | 7.53 ± 2.25 | 7.52 ± 1.69 | 8 ± 1.61 | 8.21 ± 1.4 |
| 21 | FB | 4.97 ± 1.01 | 4.84 ± 0.75 | 4.87 ± 0.62 | 5 ± 0.79 |
| 22 | FB | 8.33 ± 1.23 | 8.42 ± 0.94 | 8.4 ± 0.78 | 7.51 ± 1.28 |
| 23 | FB | 5.9 ± 2.1 | 6.36 ± 2.12 | 6.53 ± 1.76 | 6.37 ± 1.27 |
| 24 | FB | 5.03 ± 0.12 | 5.04 ± 0.33 | 5.21 ± 0.41 | 5.13 ± 0.42 |
| 25 | FB | 6.03 ± 0.45 | 5.98 ± 0.65 | 6.03 ± 0.95 | 6.45 ± 1.21 |
| 26 | FB | 8.8 ± 1.37 | 8.6 ± 1.27 | 8 ± 1.47 | 7.94 ± 1.27 |
| 27 | FB | 2.7 ± 1.54 | 3.34 ± 1.46 | 3.49 ± 1.55 | 4.01 ± 1.34 |
| 28 | FB | 5.8 ± 1.83 | 6.5 ± 1.66 | 6.79 ± 1.45 | 5.93 ± 1.48 |
| 29 | FB | 8.2 ± 1.5 | 8.46 ± 1.21 | 8.64 ± 1.04 | 8.85 ± 1 |
| 30 | FB | 6.73 ± 0.98 | 6.86 ± 0.72 | 7.4 ± 1.1 | 6.62 ± 1.56 |

1. Group C (11-18 years)

| Subjects | Breathing  methods | 3 | 5 | 7 | 15 |
| --- | --- | --- | --- | --- | --- |
| 1 | FB | 6.4 ± 0 | 6.4 ± 0 | 6.4 ± 0 | 6.45 ± 0.09 |
|  | BH | 5.8 ± 0 | 5.8 ± 0 | 5.77 ± 0.05 | 5.73 ± 0.05 |
| 2 | FB | 7.3 ± 0 | 7.3 ± 0 | 7.3 ± 0 | 6.35 ± 1.26 |
|  | BH | 5.6 ± 0 | 5.6 ± 0 | 5.6 ± 0 | 5.65 ± 0.14 |
| 3 | FB | 7.67 ± 0.06 | 7.68 ± 0.04 | 7.7 ± 0.06 | 7.92 ± 0.36 |
| 4 | FB | 4.83 ± 0.06 | 4.82 ± 0.04 | 4.81 ± 0.04 | 4.83 ± 0.08 |
|  | BH | 4.7 ± 0 | 4.7 ± 0 | 4.8 ± 0.26 | 5.12 ± 0.35 |
| 5 | FB | 6.2 ± 0 | 6.2 ± 0.07 | 6.03 ± 0.42 | 5.75 ± 0.55 |
| 6 | FB | 4.53 ± 0.31 | 5.2 ± 0.94 | 5.49 ± 0.91 | 5 ± 0.91 |
|  | BH | 6.5 ± 0.1 | 6.38 ± 0.18 | 6.4 ± 0.17 | 6.23 ± 0.4 |
| 7 | FB | 7 ± 0 | 7 ± 0 | 6.86 ± 0.24 | 7.23 ± 1.1 |
|  | BH | 8.07 ± 0.12 | 7.84 ± 0.48 | 7.6 ± 0.57 | 7.78 ± 0.5 |
| 8 | FB | 4.7 ± 0 | 4.7 ± 0 | 4.69 ± 0.04 | 4.9 ± 0.42 |
|  | BH | 5.3 ± 0 | 5.3 ± 0 | 5.3 ± 0 | 5.17 ± 0.21 |
| 9 | FB | 4.8 ± 0 | 4.8 ± 0 | 4.8 ± 0 | 5.12 ± 0.41 |
|  | BH | 6.3 ± 0 | 6.24 ± 0.13 | 6.21 ± 0.15 | 5.78 ± 0.45 |
| 10 | FB | 6.73 ± 0.92 | 6.66 ± 0.71 | 6.73 ± 0.59 | 6.58 ± 0.66 |
|  | BH | 7.2 ± 0 | 7.2 ± 0 | 7.24 ± 0.11 | 7.55 ± 0.43 |
| 11 | FB | 5.7 ± 0 | 5.44 ± 0.58 | 5.14 ± 0.69 | 4.53 ± 0.84 |
|  | BH | 5.5 ± 0 | 5.5 ± 0 | 5.47 ± 0.05 | 5.46 ± 0.05 |
| 12 | FB | 5.2 ± 0 | 5.2 ± 0 | 5.2 ± 0 | 5.18 ± 0.04 |
|  | BH | 6 ± 0 | 6 ± 0 | 6 ± 0 | 5.67 ± 0.58 |
| 13 | FB | 9.3 ± 0 | 9.3 ± 0 | 9.3 ± 0 | 9.15 ± 0.23 |
|  | BH | 9.2 ± 0 | 9.2 ± 0 | 9.09 ± 0.2 | 8.08 ± 0.99 |
| 14 | FB | 4.03 ± 0.23 | 3.98 ± 0.18 | 3.96 ± 0.15 | 4.09 ± 0.29 |
|  | BH | 4.3 ± 0 | 3.98 ± 0.44 | 3.81 ± 0.46 | 3.58 ± 0.38 |
| 15 | FB | 4.27 ± 0.12 | 4.24 ± 0.09 | 4.2 ± 0.12 | 4.29 ± 0.51 |
|  | BH | 5.9 ± 0 | 5.9 ± 0 | 5.9 ± 0 | 5.73 ± 0.28 |
| 16 | FB | 6.33 ± 0.46 | 5.96 ± 0.61 | 5.93 ± 0.51 | 5.99 ± 0.58 |
|  | BH | 5.4 ± 0 | 5.4 ± 0 | 5.39 ± 0.04 | 5.38 ± 0.07 |
| 17 | FB | 4.07 ± 0.29 | 4.04 ± 0.22 | 4.06 ± 0.18 | 4.31 ± 0.29 |
|  | BH | 4.3 ± 0 | 4.3 ± 0 | 4.3 ± 0 | 4.27 ± 0.05 |
| 18 | FB | 4.8 ± 0.35 | 4.74 ± 0.26 | 4.76 ± 0.22 | 4.91 ± 0.23 |
|  | BH | 5.4 ± 0 | 5.32 ± 0.18 | 5.23 ± 0.21 | 5.15 ± 0.22 |
| 19 | FB | 4.27 ± 0.64 | 4.22 ± 0.49 | 4.27 ± 0.41 | 4.08 ± 0.34 |
|  | BH | 4.3 ± 0 | 4.3 ± 0 | 4.41 ± 0.2 | 4.61 ± 0.26 |
| 20 | FB | 5.8 ± 0 | 5.8 ± 0 | 5.66 ± 0.68 | 5.1 ± 0.71 |
|  | BH | 4.9 ± 0 | 4.9 ± 0 | 4.9 ± 0 | 4.95 ± 0.12 |
| 21 | FB | 5.03 ± 0.06 | 4.88 ± 0.33 | 4.77 ± 0.33 | 4.49 ± 0.38 |
|  | BH | 7.3 ± 0 | 6.64 ± 0.94 | 6.6 ± 0.8 | 5.85 ± 0.95 |

1. Group D (diffuse liver disease)

| Subjects | Breathing  methods | 3 | 5 | 7 | 15 |
| --- | --- | --- | --- | --- | --- |
| 1 | FB | 8.77 ± 0.38 | 8.8 ± 0.37 | 8.71 ± 0.33 | 8.23 ± 0.75 |
| 2 | FB | 5.1 ± 0 | 5.1 ± 0 | 5.1 ± 0 | 5.25 ± 0.27 |
|  | BH | 5.4 ± 0 | 5.44 ± 0.09 | 5.49 ± 0.11 | 5.53 ± 0.12 |
| 3 | FB | 6.1 ± 0 | 6.08 ± 0.04 | 6.09 ± 0.04 | 5.92 ± 0.26 |
|  | BH | 5.4 ± 0 | 5.4 ± 0 | 5.43 ± 0.05 | 5.55 ± 0.14 |
| 4 | FB | 7.5 ± 0 | 7.56 ± 0.13 | 7.83 ± 0.62 | 7.69 ± 0.48 |
|  | BH | 7.5 ± 0 | 7.5 ± 0 | 7.47 ± 0.05 | 7.47 ± 0.05 |
| 5 | FB | 9.3 ± 0.26 | 9.22 ± 0.94 | 8.79 ± 1.07 | 7.9 ± 1.14 |
|  | BH | 7.3 ± 0 | 7.3 ± 0 | 7.3 ± 0 | 7.33 ± 0.05 |
| 6 | FB | 7.4 ± 0 | 7.34 ± 0.13 | 7.16 ± 0.4 | 7.19 ± 0.44 |
|  | BH | 6.9 ± 0 | 6.9 ± 0 | 6.9 ± 0 | 6.84 ± 0.12 |
| 7 | FB | 8.8 ± 0 | 8.78 ± 0.04 | 8.94 ± 0.28 | 9.01 ± 0.74 |
|  | BH | 9.4 ± 0 | 9.4 ± 0 | 9.43 ± 0.08 | 9.64 ± 0.35 |
| 8 | FB | 2.53 ± 0.32 | 12.48 ± 0.24 | 12.21 ± 0.49 | 13.17 ± 1.2 |
| 9 | FB | 6.3 ± 0 | 6.3 ± 0 | 6.27 ± 0.05 | 6.27 ± 0.44 |
|  | BH | 8.3 ± 0 | 8.3 ± 0 | 8.3 ± 0 | 8.09 ± 0.23 |
| 10 | FB | 5.77 ± 0.23 | 5.6 ± 0.3 | 5.69 ± 0.37 | 6.21 ± 0.61 |
|  | BH | 8.7 ± 0 | 8.54 ± 0.36 | 8.23 ± 0.66 | 8.78 ± 1.66 |
| 11 | FB | 3.2 ± 0.53 | 13.62 ± 0.69 | 14.13 ± 1.03 | 13.27 ± 1.26 |
|  | BH | 8.47 ± 0.75 | 8.08 ± 0.75 | 8.06 ± 0.71 | 10.55 ± 2.98 |
| 12 | FB | 8.1 ± 0 | 8.16 ± 0.13 | 8.3 ± 0.3 | 8.17 ± 0.46 |
|  | BH | 6.6 ± 0 | 6.6 ± 0 | 6.83 ± 0.39 | 7.57 ± 0.85 |

The values are presented as mean ± standard deviation in the unit of kPa.

FB, free breathing; BH, breath holding
